# Spirituality, Culture and Job Satisfaction in the Holistic Assessment of Nurses’ Well‐Being at Work: A Cross‐Sectional Survey Study

**DOI:** 10.1155/jonm/4922972

**Published:** 2025-12-30

**Authors:** Sabina Ličen, Mirko Prosen

**Affiliations:** ^1^ Department of Nursing, Faculty of Health Sciences, University of Primorska, Izola, Slovenia, upr.si

**Keywords:** job satisfaction, nurses, occupational health, positive practice environment, workplace well-being

## Abstract

**Background:**

Nurses’ well‐being at work has become a global concern due to persistent stressors such as staff shortages, shift work, emotional demands and limited autonomy. Most existing studies take a deficit‐centred perspective, focussing on burnout or work stress and ignoring the complexity of holistic well‐being.

**Aim:**

The aim of this study was to assess nurses’ levels of well‐being at work across six well‐being domains and explore its relationship with job satisfaction, health status and functional limitations.

**Methods:**

A cross‐sectional survey was conducted among 767 nurses working in various healthcare institutions in Slovenia. The six‐dimensional well‐being scale for nurses, which was translated and psychometrically validated for this study, was used to assess physical, emotional, intellectual, social, occupational and spiritual well‐being.

**Results:**

The overall median score for nurses’ well‐being at work was 2.97 (on a scale of 1–5), slightly below the neutral midpoint (*M* = 3.00). Among the dimensions, physical (*M* = 3.12), emotional (*M* = 3.18) and spiritual (*M* = 3.21) were rated lowest, while social (*M* = 3.60) and occupational (*M* = 3.58) were rated highest. Nurses with long‐term health conditions or activity limitations gave significantly lower scores for well‐being in the physical, emotional and occupational domains (*p* < 0.001). Higher job satisfaction was positively correlated with general well‐being (*r*_*s*_ = 0.327, *p* < 0.001). Age and length of service were weakly negatively correlated with several domains of well‐being (*p* < 0.001).

**Conclusions:**

Nurses’ well‐being at work has many facets and is influenced by personal health, job satisfaction and professional context. A holistic model can highlight strengths and weaknesses that are often overlooked in traditional, burnout‐centred approaches.

**Implications for Nursing Management:**

Nurse managers should incorporate well‐being into HR policy through relationship‐centred leadership, flexible working practises and values‐led support. Interventions should include reflective spaces, mentoring and learning opportunities tailored to emotional, intellectual and spiritual development. Monitoring dimensions of well‐being as a key performance measure can lead to sustainable improvements in healthcare.

## 1. Introduction

Nurses are the backbone of the healthcare system worldwide, providing complex and emotionally demanding care in often difficult conditions [1]. However, persistent stressors such as staff shortages, increasing workloads, emotional labour, administrative burdens and workplace violence have contributed to a decline in the well‐being of nurses globally [2–4]. The consequences are far‐reaching: reduced quality of care, increased turnover intentions, absenteeism and negative psychological outcomes such as burnout, anxiety and disengagement [5, 6].

Nurses’ occupational well‐being is generally understood as a subjective evaluation of their own experiences at work, encompassing emotional, cognitive, social and existential aspects [5]. Despite increasing recognition of its importance, many existing studies have focussed on isolated constructs such as stress, job satisfaction or burnout, without adequately addressing the complex and interdependent nature of well‐being [7, 8]. The measurement tools used often reflect this fragmented approach, lacking a clear theoretical foundation or holistic scope [8].

To address this gap, scholars have recently called for the adoption of broader conceptual frameworks that reflect the multidimensional and dynamic nature of well‐being in healthcare professions [6, 7, 9]. One such framework is the six dimensions of wellness model developed by Dr Bill Hettler [10], which provides a comprehensive approach to well‐being by integrating six interrelated dimensions: physical, emotional, intellectual, social, spiritual and environmental. While the model has been widely used in the development of wellness programmes, it remains underutilised in empirical nursing research. This underuse may be attributed to the historical dominance of deficit‐oriented approaches in occupational health, which have traditionally emphasised dysfunction (e.g. stress and burnout) rather than holistic well‐being. Recent events, particularly the COVID‐19 pandemic, have shown the limitations of such narrow perspectives and renewed interest in models that consider the full human experience of healthcare work [4, 11].

Yu et al. [12] show that the general well‐being of nurses is influenced not only by job‐related stress but also by personality traits, level of anxiety, marital status and perceived workplace relationships. Their results showed that anxiety and interpersonal stress, particularly stress from colleagues, were significantly associated with lower well‐being scores. Similarly, Wood et al. [13] found that job satisfaction and psychological well‐being of advanced practice nurses varied by role, autonomy and support, suggesting that workplace structures need to take personal and contextual differences into account when promoting nurses’ well‐being. These findings are consistent with a multidimensional approach to well‐being that incorporates both internal dispositions and external working conditions.

Hettler’s model (1976) is especially relevant in this context, as it encourages a systemic and person‐centred approach to workplace health, aligning organisational goals with the broader needs of healthcare staff. Spiritual well‐being, for example, has been linked to greater compassion and resilience in high‐pressure environments such as surgical and critical care units[11], while emotional and social well‐being have been shown to influence nurses’ work engagement, retention and psychological safety [5]. Integrating these domains into a unified model allows for a more nuanced understanding of what it means to thrive in the healthcare workplace.

The aim of this cross‐sectional study is to measure nurses’ workplace well‐being across Hettler’s six dimensions and to explore the differences between them. In doing so, we aim to provide empirical evidence that can support the development of comprehensive and targeted strategies for promoting the holistic well‐being of nurses across diverse healthcare settings. To achieve these aims, the study addresses the following research questions.

1. What is the overall level of perceived well‐being at work among nurses in Hettler’s six dimensions?
2. How does the nurses’ perceived level of well‐being in Hettler’s six dimensions relate to their self‐rated health, the presence of long‐term illnesses and health‐related activity limitations?
3. To what extent is the nurses’ perceived level of well‐being at work in the six dimensions of the Hettler model related to their job satisfaction?

## 2. Methods

### 2.1. Design and Settings

This cross‐sectional study was carried out using an online survey conducted in various healthcare facilities in Slovenia. In order to ensure geographical representativeness within the relatively small national territory, the link to the survey was first distributed to designated contact nurses in various healthcare facilities in all regions of Slovenia. These contact nurses were asked to snowball the survey link among their colleagues, thus increasing the reach of the invitation within the professional nursing community.

### 2.2. Participants

The target population of this study comprised all nurses employed in Slovenia. According to the National Institute of Public Health [14], there were 22,576 registered nurses in the country in 2023. A purposive sampling method was used to recruit participants who were actively working in the nursing profession at the time of data collection. The data was collected using an anonymous, web‐based, self‐administered questionnaire. The survey link was accessed a total of 1489 times. Of these, 74 responses were excluded due to incomplete data, resulting in a final analytical sample of 767 completed questionnaires.

In order to assess the adequacy of the sample, several calculations were performed on power and sample size. For population‐based proportion estimation, a minimum sample size of 378 was required to estimate an outcome proportion of 50 with a margin of error of 5% and a confidence level of 95, assuming a population size of 22,576 and a design effect (DEFF) of 1. Our final sample of 767 clearly exceeds this threshold and ensures statistical reliability for frequency‐based analyses. In addition, G∗Power 3.1 was used to perform a power analysis for a one‐way ANOVA with independent groups. Assuming a mean effect size (*f* = 0.25), *α* = 0.05 and power = 0.95, the total sample size required was 305 participants. With a final sample size of 767, our study has more than sufficient power to detect statistically significant group differences between comparison groups. These analyses confirm that the achieved sample size is adequate and sufficiently powerful for the descriptive and inferential statistical analyses used in this study.

### 2.3. Instrument

The instrument used in this study was a structured questionnaire developed on the basis of the six dimensions of well‐being model [10], which conceptualises well‐being as a holistic and multidimensional construct comprising six interrelated domains: physical, emotional, intellectual, social, occupational and spiritual well‐being. This model has been adapted to the realities of the nursing profession and used to assess the job‐related well‐being of nurses in different healthcare settings. Each dimension captures a specific aspect of the way nurses experience and maintain their well‐being in their professional environment. Physical well‐being refers to health behaviours and energy management in the context of demanding clinical work, including rest, nutrition and physical resilience. Emotional well‐being reflects the ability to manage stress, maintain a positive attitude and deal with emotionally intense situations that are common in nursing. Intellectual well‐being includes opportunities for professional growth, critical thinking and continuous learning within the healthcare system. Social well‐being includes collegial support, effective teamwork and good interpersonal relationships with colleagues, patients and other professionals. Occupational well‐being refers to the alignment between personal values and professional responsibilities, job satisfaction and the perceived meaning and fulfilment of nursing work. Spiritual well‐being includes the search for meaning and purpose in nursing and the congruence between personal beliefs and daily professional practice [10]. The resulting measurement tool, the six‐dimensional wellness scale for nurses, consisted of 30 items, with five statements representing each dimension. Participants rated their agreement with each item using a five‐point Likert scale ranging from 1 (strongly disagree) to 5 (strongly agree).

To determine content validity, a panel of six experts, two men and four women with at least 5 years of experience in nursing practice or nursing management (mean age = 44 years; SD = 8.76) were asked to rate the clarity and relevance of each item. The content validation process was conducted with the experts rating the items independently using a standardised content validation form. Each item was rated on a four‐point relevance scale (1 = not relevant to 4 = very relevant), and item‐level indices of content validity (I‐CVI) were calculated as the proportion of experts who rated the item as 3 or 4. The CVI at scale level (S‐CVI/Ave) was calculated as the average of all I‐CVI values. Following best‐practice recommendations [15, 16], a cut‐off value of 0.83 was used to determine adequate content validity. All 30 items achieved I‐CVI values between 0.83 and 1.00, and the overall S‐CVI/Ave for the scale was 0.94, indicating excellent content validity. No items were deleted. However, minor wording adjustments were made based on expert feedback to improve clarity while maintaining the intended meaning.

Internal consistency was excellent for the overall scale (Cronbach’s *α* = 0.945). The reliability coefficients of the subscales were also acceptable to excellent: Physical (*α* = 0.724), Emotional (*α* = 0.905), Intellectual (*α* = 0.860), Social (*α* = 0.823), Occupational (*α* = 0.821) and Spiritual dimensions (*α* = 0.803).

To confirm the hypothesised six‐factor structure, a confirmatory factor analysis was conducted. All factor loadings and covariances between the latent factors were statistically significant (*p* < 0.001), with correlations between factors ranging from 0.268 to 0.926 (Table 1). The goodness‐of‐fit indices of the model showed an acceptable fit: *χ* ^2^(390) = 2465, *p* < 0.001; CFI = 0.886; TLI = 0.862; RMSEA = 0.0685 (90% CI = 0.0648–0.0732). While the chi‐square test was significant, as expected with a large sample, the CFI and TLI values approached conventional thresholds for goodness of fit, and the RMSEA value was within acceptable limits. These results confirm the appropriateness of the six‐factor model, but at the same time suggest that further refinement or cross‐validation could improve the performance of the model.

**Table 1:** Dimensions covariances in the structural equation model.

Dimensions	Estimate	Std. error	95% CI		*z*‐value	*p*
			Lower	Upper		
Physical dimension of well‐being ↔ intellectual dimension of well‐being	0.428	0.045	0.339	0.517	9.44	*< 0.001*
Physical dimension of well‐being ↔ emotional dimension of well‐being	0.327	0.047	0.235	0.419	6.95	*< 0.001*
Physical dimension of well‐being ↔ spiritual dimension of well‐being	0.589	0.044	0.504	0.674	13.53	*< 0.001*
Physical dimension of well‐being ↔ social dimension of well‐being	0.354	0.049	0.258	0.451	7.21	*< 0.001*
Physical dimension of well‐being ↔ occupational dimension of well‐being	0.268	0.056	0.158	0.377	4.78	*< 0.001*
Intellectual dimension of well‐being ↔ emotional dimension of well‐being	0.926	0.013	0.901	0.952	71.69	*< 0.001*
Intellectual dimension of well‐being ↔ spiritual dimension of well‐being	0.772	0.027	0.719	0.825	28.54	*< 0.001*
Intellectual dimension of well‐being ↔ social dimension of well‐being	0.909	0.017	0.876	0.942	53.92	*< 0.001*
Intellectual dimension of well‐being ↔ occupational dimension of well‐being	0.695	0.032	0.630	0.760	21.01	*< 0.001*
Emotional dimension of well‐being ↔ spiritual dimension of well‐being	0.731	0.028	0.676	0.0786	26.12	*< 0.001*
Emotional dimension of well‐being ↔ social dimension of well‐being	0.872	0.018	0.837	0.907	48.38	*< 0.001*
Emotional dimension of well‐being ↔ occupational dimension of well‐being	0.588	0.036	0.517	0.659	16.25	*< 0.001*
Spiritual dimension of well‐being ↔ social dimension of well‐being	0.849	0.023	0.803	0.895	36.33	*< 0.001*
Spiritual dimension of well‐being ↔ social dimension of well‐being	0.680	0.039	0.603	0.758	17.20	*< 0.001*
Social dimension of well‐being ↔ occupational dimension of well‐being	0.751	0.031	0.690	0.811	24.27	*< 0.001*

*Note:* Rating is based on five response categories, ranging from 1–strongly disagree to 5–strongly agree.

Higher median scores on each subscale reflect higher perceived well‐being within that specific dimension, while lower scores indicate lower perceived well‐being. The same interpretation applies to the overall scale: a higher overall median score indicates greater holistic well‐being in the workplace.

### 2.4. Data Collection

Data was collected via the secure online survey platform 1ka.arnes.si, which was hosted on an academic server. Access to the raw data was restricted to the principal investigator only to ensure confidentiality and integrity of the data throughout the research process. Participation in the study was voluntary and respondents were assured complete anonymity. Before starting the questionnaire, participants received detailed information about the purpose of the study, data protection measures and instructions for completing the survey. Informed consent was obtained digitally via a consent checkbox (“I agree to participate”) at the beginning of the survey. Participants were informed that they could cancel their participation at any time without negative consequences.

### 2.5. Data Analysis

All statistical analyses were performed with IBM SPSS version 29.0 (SPSS Inc., Chicago, IL, USA) and Jamovi version 2.3, an open‐source statistical software. The internal consistency of the six‐dimensional well‐being scale for nurses and its subscales was assessed using Cronbach’s alpha coefficients. Confirmatory factor analysis (CFA) was performed to test the hypothesised six‐dimensional structure of the scale and model fit was assessed using several indices: *χ* ^2^, RMSEA, CFI and TLI. Prior to the inferential tests, the distribution of well‐being scores was analysed using the Shapiro–Wilk test. As all six well‐being dimensions and the total score deviated significantly from a normal distribution (*p* < 0.001), nonparametric statistical tests were used. The Wilcoxon signed‐rank test was used to compare the median values of the individual dimensions of well‐being and the overall scale with the theoretical median of 3.00, which represents a neutral response on the five‐point Likert scale. Mann–Whitney *U*‐tests for binary variables (presence vs. absence of long‐term health conditions) and Kruskal–Wallis H‐tests for ordinal variables with more than two groups (general health status, activity limitations, job satisfaction) were used to assess group differences. Spearman’s rho was used to analyse associations between continuous/ordinal variables (age and seniority) and the six dimensions of well‐being as well as the total well‐being score. A two‐sided significance level of *p* < 0.05 was used for all inferential analyses.

### 2.6. Ethical Considerations

Ethical approval for this study was granted by the Commission of the University of Primorska for Ethics in Human Subjects Research (approval number: 4264‐16‐3/2022). The survey complied with the EU General Data Protection Regulation (GDPR) on data protection. Only the principal investigator had access to the raw data. Participants also had the right to skip questions they did not want to answer and could withdraw from the study at any time without consequences. This study was reported in accordance with the STROBE (Strengthening the Reporting of Observational Studies in Epidemiology) checklist for cross‐sectional studies.

## 3. Results

The final sample consisted of 767 nurses, of whom 89 (11.60%) were men and 678 (88.40%) women. The participants were between 21 and 64 years old, with a mean age of 33.19 years (SD = 9.91). The average length of work experience was 10.57 years (SD = 10.04) and ranged from 0 to 44 years. Further characteristics of the sample can be found in Table 2.

**Table 2:** Demographic characteristics and background of the participants (*n* = 767).

Variable		*n*	%
Education	Secondary‐level educated nurse (comparable to healthcare assistant)	301	39.20
	Registered nurse with a bachelor’s degree in nursing	436	56.80
	Master’s‐prepared nurse/nurse with a master’s degree	30	3.90
Professional working environment	Hospital ward	744	97.00
	Primary health care centre/community health services/home visiting nursing	14	1.80
	Long‐term care setting	9	1.20
Self‐assessed general state of health	Very good	154	20.10
	Good	402	52.40
	Fair	145	18.90
	Poor	37	4.80
	Very poor	29	3.80
Prevalence of long‐term health conditions	Yes	241	31.40
	No	526	68.60
Extent of limitation in usual activities due to health problems in the past 6 months or more	Severely limited	19	2.50
	Moderately limited	314	40.90
	Not limited at all	434	56.60
Overall job satisfaction among participants	Very satisfied	65	8.50
	Satisfied	350	23.10
	Neither satisfied nor dissatisfied	177	45.60
	Dissatisfied	142	18.50
	Very dissatisfied	33	4.30

The majority of participants (56.80%) were registered nurses with a bachelor’s degree in nursing, followed by 39.20% who had completed secondary‐level nursing education (comparable to healthcare assistants), and 3.90% who had a master’s degree in nursing. Almost all respondents (97.0%) were employed in hospital departments, while only 1.80% worked in community or primary care and 1.20% in long‐term care. In terms of self‐rated general health, 52.40% described their health as good and 20.10% as very good, while 18.9% said their health was fair and 8.60% said it was poor or very poor. A total of 31.40% of respondents stated that they had been suffering from a long‐term illness for more than 6 months. In terms of limitations in usual activities due to health problems, 40.90% said they were moderately limited and 2.50% said they were severely limited. Finally, 31.60% of participants said they were very satisfied or satisfied with their work, 18.50% dissatisfied and 4.30% very satisfied.

Normality tests using the Shapiro–Wilk test revealed statistically significant deviations from a normal distribution for all six dimensions of well‐being and for the overall scale (*p* < 0.001 for all). To investigate whether participants’ responses deviated significantly from the midpoint of the scale, Wilcoxon signed rank tests were conducted for each of the six dimensions of well‐being and for the total scale score. The theoretical median was set at 3.00, which corresponds to the neutral category on the five‐point Likert scale. The analysis aimed to determine whether the participants’ level of perceived well‐being was significantly above or below the neutral reference point in each domain and overall. The results of the Wilcoxon signed rank tests are shown in Table 3.

**Table 3:** Median scores and results of the Wilcoxon signed rank test for the six‐dimensional wellness scale for nurses and its dimensions (*n* = 767).

Variables	Median	IQR	W	SE	*Z*	*p*
Physical dimension of well‐being	2.00	1.40	12,346.50	3412.41	−15.89	< 0.001
Intellectual dimension of well‐being	3.20	1.40	69,160.50	3233.91	1.94	0.049
Emotional dimension of well‐being	2.80	1.80	50,489.50	3270.43	−4.09	< 0.001
Spiritual dimension of well‐being	2.80	1.00	46,653.00	2962.23	−3.17	0.002
Social dimension of well‐being	3.40	1.20	61,478.50	2785.48	3.53	< 0.001
Occupational dimension of well‐being	3.40	1.00	74,987.00	2916.80	6.89	< 0.001
Six‐dimensional wellness scale for nurses	2.97	0.83	58,518.50	3615.56	−3.94	< 0.001

*Note:* IQR–interquartile range; *W*–Wilcoxon signed‐rank test statistic; SE–standard error; *Z*–standardised test statistic; *p*–significance level.

The results showed that the participants’ values in all six dimensions of well‐being deviated significantly from the neutral median value of 3.00, which represents a moderate level of perceived well‐being. The physical dimension in particular had a median value of 2.00 (*Z* = −15.89, *p* < 0.001), which indicates a significantly lower level of perceived physical well‐being. The emotional (*M* = 2.80, *p* < 0.001) and spiritual (*M* = 2.80, *p* = 0.002) dimensions also showed a significantly lower perceived well‐being. The intellectual dimension was slightly above the median (*M* = 3.20, *p* = 0.049), indicating a modestly higher perceived intellectual well‐being. The social and professional dimensions had significantly higher median values (both 3.40, *p* < 0.001), indicating higher perceived well‐being in the areas of interpersonal and professional fulfilment. The total score of the six‐dimensional scale for nurses’ well‐being was significantly below the neutral reference value with a median score of 2.97 (*Z* = −3.94, *p* < 0.001), indicating a slightly reduced overall perception of holistic well‐being at work among the participants.

The Mann–Whitney *U* test was used to analyse whether the presence of long‐term health conditions (lasting longer than 6 months) was associated with statistically significant differences in nurses’ perceived well‐being at work. The purpose of the analysis was to determine whether nurses with chronic health problems systematically reported lower well‐being on the six dimensions and the six‐dimensional wellness scale for nurses compared to their healthier colleagues (Table 4).

**Table 4:** Differences in well‐being at work among nurses based on the presence of long‐term health conditions (Mann–Whitney *U* test results) (*n* = 767).

Prevalence of long‐term health conditions	D1	D2	D3	D4	D5	D6	Scale
	M (IQR)	M (IQR)	M (IQR)	M (IQR)	M (IQR)	M (IQR)	M (IQR)
Yes	2.00 (1.00)	3.00 (160)	2.60 (1.60)	2.80 (1.40)	3.00 (1.40)	3.20 (1.20)	2.70 (1.00)
No	2.00 (1.60)	3.40 (1.20)	3.00 (2.00)	2.80 (0.80)	3.40 (0.80)	3.40 (0.80)	3.10 (0.70)
*U*	30,713.0	25,230.0	22,755.0	23,960.5	21,068.0	24,134.5	25,599.5
*Z*	−0.804	−3.816	−5.238	−1.741	−3.646	−1.534	−3.825
*p*	0.421	< 0.001	< 0.001	0.082	< 0.001	0.125	< 0.001

*Note:* *M*–median; IQR–interquartile range; *U*–Mann–Whitney test statistic; *Z*–standardised test statistic; *p*–significance level; D1–Physical dimension of well‐being; D2–Intellectual dimension of well‐being; D3–Emotional dimension of well‐being; D4–Spiritual dimension of well‐being; D5–Social dimension of well‐being; D6–Occupational dimension of well‐being; Scale–Six‐dimensional wellness scale for nurses.

The results showed significant differences in well‐being at work between nurses with and without long‐term health problems on several dimensions of the six‐dimensional wellness scale for nurses. Nurses with chronic health conditions reported significantly lower well‐being in the intellectual dimension (*M* = 3.00, IQR = 1.60) compared to those without (*M* = 3.40, IQR = 1.20), *U* = 25,230.0, *z* = −3.82, *p* < 0.001; emotional dimension (*M* = 2.60, IQR = 1.60 vs. *M* = 3.00, IQR = 2.00), *U* = 22,755.0, *z* = −5.24, *p* < 0.001; and social dimension (*M* = 3.00, IQR = 1.40 vs. *M* = 3.40, IQR = 0.8), *U* = 21,068.0, *z* = −3.65, *p* < 0.001. The total well‐being score was also significantly lower in nurses with chronic conditions (*M* = 2.70, IQR = 1.00) compared to their healthier counterparts (*M* = 3.10, IQR = 0.70), *U* = 25,599.5, *z* = −3.83, *p* < 0.001. No statistically significant differences were observed in the physical (*p* = 0.421), spiritual (*p* = 0.082), or occupational (*p* = 0.125) dimensions.

A Kruskal–Wallis *H* test was carried out to further investigate how the nurses’ self‐assessed general state of health was related to their perceived well‐being at work (Table 5). The analysis compared well‐being scores across five health categories (very good, good, fair, poor and very poor) to determine whether nurses who rated their general health worse also reported systematically lower scores on Hettler’s six dimensions and the six‐dimensional wellness scale for nurses.

**Table 5:** Differences in nurses’ well‐being at work according to self‐assessed general state of health (results of the Kruskal–Wallis *H*‐test) (*n* = 767).

Self‐assessed general state of health	D1	D2	D3	D4	D5	D6	Scale
	M (IQR)	M (IQR)	M (IQR)	M (IQR)	M (IQR)	M (IQR)	M (IQR)
Very good	2.20 (0.90)	3.40 (1.00)	3.20 (1.80)	4.00 (1.40)	3.60 (0.60)	4.00 (1.60)	3.30 (1.10)
Good	2.20 (1.60)	3.00 (1.40)	2.80 (1.40)	2.80 (1.40)	3.40 (1.80)	3.40 (1.50)	3.10 (0.50)
Fair	2.00 (1.20)	3.00 (2.20)	3.00 (3.6)	2.80 (0.80)	3.20 (1.60)	3.40 (0.70)	2.80 (1.00)
Poor	2.00 (1.40)	2.80 (1.60)	2.60 (1.4)	2.60 (1.40)	3.00 (1.40)	3.40 (1.20)	2.60 (1.00)
Very poor	1.80 (1.60)	1.90 (1.60)	1.40 (1.7)	2.20 (1.60)	2.00 (1.40)	2.60 (2.10)	2.00 (1.00)
*H*	8.016	45.841	55.533	20.595	35.062	9.459	42.515
*df*	4	4	4	4	4	4	4
*p*	0.091	< 0.001	< 0.001	0.323	0.002	< 0.051	< 0.001

*Note:* *M*–median; IQR–interquartile range; *H*–Kruskal–Wallis test statistic; *df*–degrees of freedom; *p*–significance level; D1–Physical dimension of well‐being; D2–Intellectual dimension of well‐being; D3–Emotional dimension of well‐being; D4–Spiritual dimension of well‐being; D5–Social dimension of well‐being; D6–Occupational dimension of well‐being; Scale–Six‐dimensional wellness scale for nurses.

Results showed statistically significant differences in nurses’ perceived well‐being at work across self‐assessed general health categories in five of six dimensions, as well as across the six‐dimensional wellness scale. Significant group differences were observed in the intellectual (*H* = 45.84, *p* < 0.001), emotional (*H* = 55.53, *p* < 0.001), spiritual (*H* = 20.60, *p* < 0.001), social (*H* = 35.06, *p* < 0.001) and general well‐being (*H* = 42.52, *p* < 0.001) domains. In each of these areas, participants with poorer self‐assessed health reported consistently lower median well‐being scores. For example, emotional well‐being dropped from a median score of 3.2 (IQR = 1.80) for participants with very good health to 1.4 (IQR = 1.70) for participants with very poor health. The occupational dimension (*H* = 9.46, *p* = 0.051) and the physical dimension (*H* = 8.02, *p* = 0.091) did not reach statistical significance.

A Kruskal–Wallis *H* test was also conducted to investigate the relationship between health‐related activity limitations and nurses’ well‐being at work (Table 6). The grouping variable was the self‐reported extent to which they had been restricted in their usual activities in the last 6 months due to health problems, divided into three categories: severely limited, moderately limited and not limited at all.

**Table 6:** Differences in the well‐being of nurses at work depending on the degree of health‐related activity restriction (results of the Kruskal–Wallis *H*‐test) (*n* = 767).

Health‐related activity restriction	D1	D2	D3	D4	D5	D6	Scale
	M (IQR)	M (IQR)	M (IQR)	M (IQR)	M (IQR)	M (IQR)	M (IQR)
Severely limited	1.80 (2.40)	2.00 (2.40)	2.00 (1.60)	2.80 (1.80)	2.40 (1.60)	2.40 (2.20)	2.30 (1.50)
Moderately limited	2.00 (1.20)	3.30 (1.50)	2.60 (1.40)	3.00 (1.40)	3.20 (1.40)	3.20 (1.20)	2.80 (1.00)
Not limited at all	2.20 (1.40)	3.40 (1.20)	3.30 (1.80)	2.60 (0.70)	3.60 (0.60)	3.40 (0.40)	3.10 (5.50)
*H*	10.428	32.584	38.549	2.258	12.474	28.402	15.789
df	2	2	2	2	2	2	2
*p*	*0.005*	*< 0.001*	*< 0.001*	*0.323*	*0.002*	*< 0.001*	*< 0.001*

*Note:* *M*–median; IQR–interquartile range; *H*–Kruskal–Wallis test statistic; df–degrees of freedom; *p*–significance level; D1–Physical dimension of well‐being; D2–Intellectual dimension of well‐being; D3–Emotional dimension of well‐being; D4–Spiritual dimension of well‐being; D5–Social dimension of well‐being; D6–Occupational dimension of well‐being; Scale–Six‐dimensional wellness scale for nurses.

The Kruskal–Wallis *H*‐test showed statistically significant differences in nurses’ perceived well‐being at work based on the degree of health‐related activity limitation during the last 6 months. In particular, significant effects were observed for the dimensions of physical (*H* = 10.43, *p* = 0.005), intellectual (*H* = 32.58, *p* < 0.001), emotional (*H* = 38.55, *p* < 0.001), social (*H* = 12.47, *p* = 0.002), occupational (*H* = 28.40, *p* < 0.001) and general well‐being (*H* = 15.79, *p* < 0.001). In each of these dimensions, nurses who reported being severely limited by health problems consistently reported the lowest well‐being scores. No significant difference was found in the spiritual dimension (*H* = 2.26, *p* = 0.323).

A Kruskal–Wallis *H*‐test was conducted to analyse the relationship between nurses’ general job satisfaction and their perceived well‐being at work (Table 7). Job satisfaction was assessed using a five‐point ordinal scale ranging from very dissatisfied to extremely satisfied, and participants were grouped accordingly.

**Table 7:** Differences in the well‐being of nursing staff at work according to the degree of job satisfaction (results of the Kruskal–Wallis *H*‐test) (*n* = 767).

Job satisfaction	D1	D2	D3	D4	D5	D6	Scale
	M (IQR)	M (IQR)	M (IQR)	M (IQR)	M (IQR)	M (IQR)	M (IQR)
Very dissatisfied	1.80 (0.80)	2.40 (1.10)	2.00 (1.80)	2.20 (1.60)	2.20 (1.00)	3.00 (1.50)	2.30 (1.00)
Dissatisfied	1.90 (1.60)	2.60 (1.40)	2.00 (1.50)	2.60 (1.30)	2.60 (1.20)	3.20 (1.00)	2.40 (1.00)
Neither satisfied nor dissatisfied	2.00 (1.20)	3.20 (1.20)	2.80 (1.20)	2.80 (0.80)	3.40 (1.00)	3.40 (1.60)	2.90 (0.80)
Satisfied	2.20 (1.20)	3.40 (1.10)	3.30 (1.20)	3.20 (1.00)	3.60 (0.60)	3.40 (0.60)	3.10 (0.50)
Very satisfied	2.60 (1.00)	3.80 (0.80)	3.60 (1.40)	3.50 (1.00)	3.90 (0.40)	3.80 (0.70)	3.40 (0.60)
*H*	12.523	119.737	111.834	49.777	101.425	20.694	98.805
df	4	4	4	4	4	4	4
*p*	0.014	< 0.001	< 0.001	< 0.001	< 0.001	< 0.001	< 0.001

*Note:* *M*–median; IQR–interquartile range; *H*–Kruskal–Wallis test statistic; df–degrees of freedom; *p*–significance level; D1–Physical dimension of well‐being; D2–Intellectual dimension of well‐being; D3–Emotional dimension of well‐being; D4–Spiritual dimension of well‐being; D5–Social dimension of well‐being; D6–Occupational dimension of well‐being; Scale–Six‐dimensional wellness scale for nurses.

The Kruskal–Wallis *H*‐test indicated statistically significant differences in all six dimensions of well‐being at work and in the overall scale based on nurses’ job satisfaction. Higher job satisfaction was consistently associated with higher perceived well‐being. For example, emotional well‐being increased from a median score of 2.0 (IQR = 1.50) for the very dissatisfied nurses to 3.6 (IQR = 1.40) for the very satisfied nurses, *H* = 111.83, *p* < 0.001. Similar trends were observed for intellectual well‐being (*H* = 119.74, *p* < 0.001), spiritual well‐being (*H* = 49.78, *p* < 0.001), social well‐being (*H* = 101.43, *p* < 0.001) and occupational well‐being (*H* = 20.69, *p* < 0.001). The six‐dimensional scale for Nurses’ well‐being also showed significant differences in terms of job satisfaction, with the median score ranging from 2.3 (IQR = 1.00) for very dissatisfied nurses to 3.4 (IQR = 0.60) for very satisfied nurses (*H* = 98.81, *p* < 0.001). There were also significant, albeit smaller, differences in the physical dimension (*H* = 12.52, *p* = 0.014).

The correlation coefficients were calculated using Spearman’s rho in order to analyse the relationships between the demographic variables and perceived well‐being at work (Table 8).

**Table 8:** Spearman’s correlations between age, length of service, job satisfaction and dimensions of well‐being at work (*n* = 767).

Dimension of well‐being	Age (*r*_*s*_)	Length of service (*r*_*s*_)	Job satisfaction (*r*_*s*_)
Physical dimension of well‐being	−0.027 (ns)	0.008 (ns)	0.123^∗∗^
Intellectual dimension of well‐being	−0.165^∗∗^	−0.163^∗∗^	0.286^∗∗^
Emotional dimension of well‐being	−0.163^∗∗^	−0.156^∗∗^	0.259^∗∗^
Spiritual dimension of well‐being	−0.073 (ns)	−0.076 (ns)	0.308^∗∗^
Social dimension of well‐being	−0.131^∗∗^	−0.126^∗∗^	0.347^∗∗^
Occupational dimension of well‐being	−0.155^∗∗^	−0.172^∗∗^	0.083 (ns)
Six‐dimensional wellness scale for nurses	−0.169^∗∗^	−0.163^∗∗^	0.327^∗∗^

Abbreviation: ns, not significant.

^∗∗^Correlation is significant at the 0.001 level.

Spearman’s correlation analysis revealed statistically significant weak negative correlations between age and several dimensions of well‐being at work. Age was weakly negatively correlated with intellectual (*r*_*s*_ = −0.17, *p* < 0.001), emotional (*r*_*s*_ = −0.16, *p* < 0.001), social (*r*_*s*_ = −0.13, *p* = 0.004) and occupational well‐being (*r*_*s*_ = −0.16, *p* < 0.001) as well as with the overall six‐dimensional well‐being scale (*r*_*s*_ = −0.17, *p* < 0.001). Similarly, the duration of service was weakly negatively correlated with intellectual (*r*_*s*_ = −0.16, *p* < 0.001), emotional (*r*_*s*_ = −0.16, *p* < 0.001), social (*r*_*s*_ = −0.13, *p* = 0.006), occupational (*r*_*s*_ = −0.17, *p* < 0.001) and general well‐being (*r*_*s*_ = −0.16, *p* < 0.001). No significant correlations were observed with the physical or spiritual dimensions (*p* > 0.05). It was also found that job satisfaction correlates positively with several dimensions of well‐being at work. Statistically significant weak to moderate positive correlations were found between job satisfaction and the intellectual (*r*_*s*_ = 0.29, *p* < 0.001), emotional (*r*_*s*_ = 0.26, *p* < 0.001), spiritual (*r*_*s*_ = 0.31, *p* < 0.001) and social dimensions of well‐being (*r*_*s*_ = 0.35, *p* < 0.001). In addition, a significant positive correlation was found with the physical dimension of well‐being (*r*_*s*_ = 0.12, *p* < 0.001) and with the overall six‐dimensional scale for nurses’ well‐being (*r*_*s*_ = 0.33, *p* < 0.001), meaning that higher levels of job satisfaction are associated with higher well‐being in most domains. No statistically significant correlation was observed between job satisfaction and the occupational dimension of well‐being (*p* > 0.05).

## 4. Discussion

The aim of this study was to assess the holistic well‐being of nurses in the workplace using Hettler’s six dimensions of well‐being model. This model provides a comprehensive framework for understanding how occupational, emotional, intellectual, physical, social and spiritual factors influence well‐being in healthcare. Results indicate that while nurses reported moderate levels of overall well‐being, the median score was below the theoretical mean of the scale, suggesting an underlying strain in several dimensions of well‐being at work. In particular, the areas of emotional, spiritual and physical well‐being received the lowest scores, while the areas of social and professional well‐being were rated the highest. These results emphasise the multiple challenges nurses face and point to both protective factors and areas where targeted interventions are needed.

The relatively low scores in the emotional and spiritual domains are consistent with the international literature, which points to the ongoing emotional demands of the nursing profession [17, 18]. Emotional labour, which involves managing internal emotional states to meet professional expectations, has been consistently linked to burnout and emotional exhaustion [19]. Nurses who rely heavily on surface acting, the suppression of genuine feelings, are particularly prone to poor well‐being, including lower job satisfaction and increased turnover intentions [20]. Similarly, Fu et al. [2] show that emotion regulation mediates the relationship between work stressors and occupational well‐being, further supporting the importance of supporting nurses’ emotional skills. The findings of this study suggest that emotional well‐being may be an important focus for organisational strategies aimed at retaining and empowering nurses.

Physical well‐being was also found to be of concern, possibly due to long‐standing systemic issues such as shift work, understaffing and the physically demanding nature of clinical nursing. Comparable research has found that physical fatigue and lifestyle issues, including inadequate rest and disordered eating habits, are prevalent among nursing staff [21]. These factors not only affect individual health, but also impair professional performance and resilience. The consistently low scores in the physical domain emphasise the need for management initiatives that address workload, ergonomics and recovery time [22].

In contrast, nurses reported relatively high levels of social and occupational well‐being. These findings may reflect the strength of collegial relationships and the intrinsic sense of purpose that derives from the clinical role [23]. Wood et al. [3] emphasise that the feeling of making a difference, meaningful work and strong collegial networks are crucial for nurses’ job satisfaction. Similarly, social bonds and professional identity can act as a buffer against the negative effects of stress and burnout and provide a sense of belonging and validation. Team‐based support structures and recognising nurses’ contributions can serve as valuable protective mechanisms [24, 25].

Another important finding is the strong relationship between job satisfaction and all dimensions of well‐being at work. Nurses who reported high job satisfaction scored significantly higher in the emotional, intellectual and social domains. This is consistent with previous findings by Yu et al. [12], who reported that job satisfaction is a robust predictor of overall well‐being in the nursing population. Furthermore, self‐determination theory [7] provides a useful tool for understanding this dynamic as it emphasises that autonomy, competence and relatedness are fundamental to human well‐being. A work environment that supports these psychological needs is more likely to promote motivation, satisfaction and sustained engagement [26]. In this study, the positive interplay between satisfaction and well‐being underlines the importance of developing an organisational culture that supports needs.

Age and experience were weakly negatively associated with well‐being, suggesting that older or more tenured nurses scored slightly lower on several dimensions. Although the effect sizes were small, this trend may reflect the cumulative burden of job stressors such as emotional demands, limited autonomy and chronic understaffing, factors that have been consistently reported in nursing [2, 5]. These results are consistent with recent findings by Beier et al. [27] who found that although older nurses reported lower depersonalisation and used more positive coping strategies, they did not consistently report lower emotional exhaustion. This suggests that while experience may improve coping and reduce cynicism, it does not fully protect against the emotional exhaustion that has accumulated over time. Furthermore, long‐term exposure to stress may reduce nurses’ ability to recover, especially when support systems are inadequate or when organisational values conflict with personal beliefs [6, 13]. In addition, Wang et al. [28] found similar patterns in male nurses, noting that career stagnation and lack of recognition may contribute to declining engagement and satisfaction in more experienced cohorts. Generational differences may also play a role, as younger nurses may have different expectations, value systems, or greater flexibility in adapting to new career challenges, while older nurses may experience value dissonance or stagnation in career development [29]. Importantly, these patterns require age‐ and seniority‐dependent interventions that strengthen resources and maintain emotional vitality across careers [30].

This study makes an important contribution to the topic by operationalising Hettler’s model in a nursing context. Although the model is widely used in the fields of wellness and education, its application in nursing workforce research has been limited. The positive internal consistency of the adapted scale and the differentiated responses across dimensions support the relevance and utility of the model in assessing the complex experiences of nurses in the workplace. Furthermore, the strength of the model lies in its holistic orientation, which goes beyond deficit‐oriented approaches such as burnout alone and instead recognises the interplay of personal, interpersonal and organisational factors that influence well‐being.

Given the global shortage of nurses and rising turnover rates, understanding the multidimensional nature of well‐being is of urgent practical importance. As Xiao, Cooke and Chen [6] argue, well‐being in nursing is not just an individual matter but a systemic outcome shaped by work environments, leadership practises and broader health policies. The present findings are consistent with their call for integrated, multi‐level strategies that improve well‐being through structural support, capacity building and policy innovation. Furthermore, this study provides both empirical and theoretical insights into the state of nurses’ well‐being in the workplace. The findings identify specific areas where nurses excel and areas where they face challenges, providing a roadmap for nurse managers and policymakers to design targeted interventions that address the workforce’s holistic needs. Future research should examine these relationships longitudinally and across different healthcare settings to clarify causal pathways and evaluate the effectiveness of interventions.

## 5. Limitations of the Study

Despite its contribution, this study has several limitations that should be recognised. First, the study used a cross‐sectional design, which limits the ability to draw causal conclusions between well‐being at work and related variables such as job satisfaction or health status. Although correlations were observed, it is not possible to determine the direction of these relationships. Future research should use longitudinal or experimental designs to examine how workplace interventions or organisational changes affect the six dimensions of well‐being over time. Secondly, data collection was based solely on self‐report, which may be subject to social desirability or recall bias. Although anonymity was maintained to minimise this risk, participants may have over‐ or under‐reported their well‐being or satisfaction due to perceived expectations. The inclusion of objective indicators of well‐being, such as absenteeism, retention rates or health data, could complement self‐report and improve the robustness of future analyses. Third, the sample was limited to nurses working in a specific national context and mainly in hospitals. This limits the generalisability of the results to other professional groups, healthcare systems or outpatient nurses. To address this issue, future studies should aim to include different healthcare settings, broader professional groups (e.g. midwives) and international samples to enable cross‐cultural comparisons and improve external validity.

Although this study was conducted in Slovenia, the six‐dimensional wellness model offers a holistic framework that is conceptually transferable across cultural and healthcare settings. Future cross‐national research could examine potential differences in how nurses perceive and prioritise dimensions of well‐being. In addition, longitudinal or experimental designs are needed to track changes in nurses’ well‐being over time and to strengthen causal interpretations.

## 6. Conclusion and Implications

The results of this study highlight the need for a paradigm shift in the way nursing management conceptualises and addresses well‐being in the workplace. Rather than relying solely on deficit‐based indicators such as burnout or turnover rates, managers should take a holistic approach based on multidimensional frameworks such as Hettler’s six dimensions of well‐being. This model provides a more comprehensive understanding of nurses’ life experiences and offers a valuable diagnostic tool to identify both strengths and weaknesses across the workforce. The consistently low scores for physical, emotional and spiritual well‐being emphasise the need to move beyond superficial well‐being initiatives to integrated, systemic interventions.

For nursing managers, this means investing in comprehensive well‐being programmes that cover structural, relational and developmental areas. For example, physical well‐being could be promoted through flexible working hours where possible, rest and recovery zones on wards and protected meal breaks. Emotional well‐being should be promoted through reflection groups, peer mentoring programmes and access to nonstigmatised psychological support. In the area of spiritual well‐being, managers could introduce inclusive spaces for contemplation, opportunities for values‐based discussions in clinical supervision and recognition of the existential aspects of nursing work.

Intellectual and professional well‐being, two areas closely linked to motivation and job satisfaction, can be improved by promoting professional autonomy, embedding lifelong learning in organisational culture and involving nurses in co‐designing quality improvement initiatives. In addition, social well‐being should be actively promoted through relationship‐centred leadership, team‐building strategies and a zero‐tolerance policy for incivility or exclusion. Finally, data on well‐being should be collected regularly and reported as a key performance indicator alongside patient safety and staff retention.

Investing in the well‐being of nurses is not an afterthought, but a strategic imperative that directly impacts the quality of care, the sustainability of the workforce and the resilience of the organisation. Nurse managers must act not only as operational leaders but also as stewards and guardians of workplace culture by embedding well‐being into the daily structure of healthcare delivery.

## Conflicts of Interest

The authors declare no conflicts of interest.

## Funding

This research was funded by ARIS–Slovenian Agency for Research and Innovation within the research programme no. P5‐0454.

## Data Availability

The data are available from the corresponding author upon reasonable request as the participants were assured that it would remain confidential.

## References

[1] Saleh M. S. M. , Abd-Elhamid Z. N. , Afit Aldhafeeri N. et al., Appreciative Leadership, Workplace Belongingness, and Affective Commitment of Nurses: the Mediating Role of Job Crafting, Journal of Nursing Management. (2024) 2024, no. 1, 10.1155/2024/2311882. PMC11918758 40224827

[2] Fu Y. , Qu Ge , Sun J. , Wang C. , and Wang J. , Enhancing Occupational Well-Being Among Chinese Nurses: Exploring the Mediation of Job Stress in the Relationship Between Social Support and Occupational Well-Being, Journal of Nursing Management. (2025) 2025, no. 1, 10.1155/jonm/2140829. PMC11978472 40223898

[3] Wood E. , King R. , Robertson S. , Senek M. , Tod A. , and Ryan T. , Sources of Satisfaction, Dissatisfaction and well-being for UK Advanced Practice Nurses: a Qualitative Study, Journal of Nursing Management. (2021) 29, no. 5, 1073–1080, 10.1111/jonm.13245. 33404130

[4] Yesildag A. Y. , Kurtaran A. T. , and Sevim F. , Assessing Workplace well-being in Healthcare: the violence-prevention Climate and Its Relationship with Workplace Happiness, International Nursing Review. (2025) 72, no. 2, 10.1111/inr.13026. PMC11969316 39140147

[5] Gao X.L. , Tong Z. , MinXia Du , Ran H. , and Wang LiNa , Relationship Between Emotional Intelligence, Occupational Well-Being, and Work Engagement Among Chinese Clinical Nurses, Asian Nursing Research. (2024) 18, no. 3, 253–259, 10.1016/j.anr.2024.07.003. 39033961

[6] Xiao Q. , Cooke F. L. , and Chen L. , Nurses’ well-being and Implications for Human Resource Management: a Systematic Literature Review, International Journal of Management Reviews. (2022) 24, no. 4, 599–624, 10.1111/ijmr.12295.

[7] Hindman J. , Zugai J. , Foster K. , and Raeburn T. , Self-Determination Theory as a Framework for Research and Design of Digital Applications for Nurses′ Well-Being, Journal of Advanced Nursing n/a. (2025) 10.1111/jan.16895. PMC12721922 40098227

[8] Wati S. G. , Effendy C. , Alim S. , Martani H. R. , and Nisman W. A. , A Systematic Review of Instruments to Measure Nurses′ Well-Being in the Work Environment, Journal of Advanced Nursing. (2025) 81, no. 12, 8470–8503, 10.1111/jan.16871. 40098560

[9] Shanafelt T. , Trockel M. , Stolz S. , Murphy D. , and Bryan B. , Ten Principles to Advance Occupational well-being in Health Care Organizations, Mayo Clinic Proceedings. (2025) 100, no. 6, 995–1004, 10.1016/j.mayocp.2025.03.026. 40455193

[10] Hettler B. , Nwi-Six-Dimensions-Fact-Sheet, National Wellness Institute. (1976) https://www.lindenwood.edu/files/resources/nwi-six-dimensions-fact-sheet.pdf.

[11] Şahin K. , Sennur , and Bulbuloglu S. , The Effect of Spiritual Well-Being of Perioperative Nurses on Compassion, Journal of PeriAnesthesia Nursing. (2022) 37, no. 4, 509–514, 10.1016/j.jopan.2021.09.012. 35256253

[12] Yu J. , Song Y. , Dong H. , Su X. , and Zhang P. , Factors Associated with the General well-being of Nurses in a Tertiary Chinese Hospital: a cross-sectional Study, Journal of Nursing Management. (2020) 28, no. 3, 540–547, 10.1111/jonm.12954. 31945253

[13] Wood E. , King R. , Robertson S. et al., Advanced Practice Nurses’ Experiences and well-being: Baseline Demographics from a Cohort Study, Journal of Nursing Management. (2020) 28, no. 4, 959–967, 10.1111/jonm.13030. 32501626

[14] National Institute of Public Health , Health Data. Access to Health Data Statistics, National Institute of Public Health of Republic of Slovenia. (2023) https://nijz.si/en/.

[15] Polit D. F. and Beck C. T. , The Content Validity Index: Are You Sure You Know What′s Being Reported? Critique and Recommendations, Research in Nursing & Health. (2006) 29, no. 5, 489–497, 10.1002/nur.20147, 2-s2.0-33750143276. 16977646

[16] Yusoff M. S. B. , ABC of Content Validation and Content Validity Index Calculation, Education in Medicine Journal. (2019) 11, no. 2, 49–54, 10.21315/eimj2019.11.2.6.

[17] Rachel H. , Chiara C. , Robert K. , and Francesco S. , Spiritual Care in Nursing: an Overview of the Measures Used to Assess Spiritual Care Provision and Related Factors Amongst Nurses, Acta BioMedica: Atenei Parmensis. (2019) 90, no. Suppl 4, 44–55, 10.23750/abm.v90i4-S.8300, 2-s2.0-85064815109. PMC6625560 30977748

[18] Zhang X. , Ba L. , Xu J. et al., Analysis of the Current Status of Community Nurses’ Spiritual Care Competencies and the Factors: a Descriptive Cross-Sectional Analysis, Nursing Open. (2023) 10, no. 5, 3356–3366, 10.1002/nop2.1589. 36682049 PMC10077363

[19] Park C.-chol , Cho H. , Lee D.-gwi , and Jeon H. , Latent Profile Analysis on Korean Nurses: Emotional Labour Strategies and well-being, Journal of Advanced Nursing. (2022) 78, no. 6, 1632–1641, 10.1111/jan.15062. 34618365

[20] Toscano F. , Galanti T. , and Cortini M. , Reducing Nurses’ Emotional Exhaustion and Turnover Intentions: the Role of Prosocial Orientation and Perceived Patient Gratitude in a Moderated Mediation Model, Journal of Nursing Management. (2025) 2025, no. 1, 10.1155/jonm/4445460. PMC12097863 40406735

[21] Li D.Y. , Houghton J. D. , Li X. , Peng QiQi , Li J.Q. , and Zou W.C. , The Relationship Between Commuting Stress and Nurses’ Well-Being: considering Gender Differences, Journal of Nursing Management. (2025) 2025, no. 1, 10.1155/jonm/4414417. PMC11985249 40223879

[22] Sonnentag S. , Tay L. , and Nesher Shoshan H. , A Review on Health and Well-being at Work: More than Stressors and Strains, Personnel Psychology. (2023) 76, no. 2, 473–510, 10.1111/peps.12572.

[23] Kohnen D. , De Witte H. , Schaufeli W. B. , Dello S. , Bruyneel L. , and Sermeus W. , What Makes Nurses Flourish at Work? How the Perceived Clinical Work Environment Relates to Nurse Motivation and Well-being: a Cross-Sectional Study, International Journal of Nursing Studies. (2023) 148, 10.1016/j.ijnurstu.2023.104567. 37837704

[24] Imboden M. T. , Belonging: an Essential Human and Organizational Need, American Journal of Health Promotion. (2024) 38, no. 6, 883–897, 10.1177/08901171241255204. 38783631

[25] Weller J. M. , Mahajan R. , Fahey-Williams K. , and Webster C. S. , Teamwork Matters: Team Situation Awareness to Build High-Performing Healthcare Teams, a Narrative Review, British Journal of Anaesthesia. (2024) 132, no. 4, 771–778, 10.1016/j.bja.2023.12.035. 38310070

[26] Labrague L. J. , Al Sabei S. , Al Rawajfah O. , AbuAlRub R. , and Burney I. , Interprofessional Collaboration as a Mediator in the Relationship Between Nurse Work Environment, Patient Safety Outcomes and Job Satisfaction Among Nurses, Journal of Nursing Management. (2022) 30, no. 1, 268–278, 10.1111/jonm.13491. 34601772

[27] Beier M. E. , Cockerham M. , Branson S. , and Boss L. , Aging and Burnout for Nurses in an Acute Care Setting: the First Wave of COVID-19, International Journal of Environmental Research and Public Health. (2023) 20, no. 8, 10.3390/ijerph20085565. PMC10138881 37107847

[28] Wang L. , Li H. , Li X. et al., Current Occupational well-being Status and Protective and Risk Factors of Male Nurses in Chengdu, China: a cross-sectional Study, Nursing Open. (2022) 9, no. 3, 1700–1708, 10.1002/nop2.1194. 35170257 PMC8994956

[29] Sanner-Stiehr E. , Stevanin S. , Mikkonen S. , and Kvist T. , Job Satisfaction and Generational Nursing Characteristics Among Registered Nurses in the United States, Italy and Finland: Results of a Survey Study, Journal of Nursing Management. (2021) 29, no. 8, 2364–2373, 10.1111/jonm.13397. 34173687

[30] Montayre J. , Harris C. , Li W. , Tang (Maggie) L. , West S. , and Antoniou M. , Older Nurses and Work-Related Factors that Impact their Mental Health and Wellbeing: a Qualitative Systematic Review, Contemporary Nurse. (2024) 60, no. 5, 537–554, 10.1080/10376178.2024.2368625. 38919042

